# Does Surgical Day of Week Affect Patient Outcomes and Hospital Costs Following Lumbar Fusion?

**DOI:** 10.7759/cureus.64571

**Published:** 2024-07-15

**Authors:** Andrea H Johnson, Jane C Brennan, Parimal Rana, Justin J Turcotte, Chad Patton

**Affiliations:** 1 Orthopedics, Anne Arundel Medical Center, Annapolis, USA; 2 Orthopedic Research, Anne Arundel Medical Center, Annapolis, USA; 3 Orthopedic Surgery Research, Anne Arundel Medical Center, Annapolis, USA; 4 Orthopedic and Surgical Research, Anne Arundel Medical Center, Annapolis, USA; 5 Orthopedic Surgery, Anne Arundel Medical Center, Annapolis, USA

**Keywords:** lumbar fusion, hospital costs, non-home discharge, unplanned readmission, surgical day of week

## Abstract

Background

As the population ages, surgical intervention for degenerative spine conditions is increasing, and this causes a commiserate increase in healthcare expenditures associated with these procedures. Little research has been done on the effect of early-week versus later-week surgeries on patient outcomes, cost, and length of stay (LOS) in patients undergoing lumbar fusion surgery. The purpose of this study is to compare LOS, patient outcomes, and hospital costs between patients having surgery early in the week and later in the week.

Methods

A retrospective review of 771 patients undergoing a one-, two-, or three-level lumbar fusion from December 2020 to December 2023 at a single institution was performed. Demographics, surgical details, postoperative outcomes and cost were compared between patients who had surgery on Monday, Tuesday, and Wednesday, to those having surgery Thursday or Friday. Univariate and multivariate analyses were performed to compare the groups.

Results

There were no differences in age, sex, BMI, race, American Society of Anesthesiology (ASA) scores, Charlson Comorbidity Index (CCI) scores, number of operative levels or inpatient/outpatient status between early- and late-week surgeries. Postoperatively the only significant difference was cost, late-week surgeries were, on average, $3,697 more expensive than early-week surgeries ($26,506 vs. $22,809; p<0.001). On multivariate analysis late-week surgeries were 2.47 times more likely to have a non-home discharge (OR: 2.47, 95% CI: 1.24 to 4.95; p=0.010) and 2.19 times more likely to have a 30-day readmission (OR: 2.19, 95% CI:1.01 to 4.74; p=0.044) Additionally, late-week surgeries were $2,041.55 (β:2,041.55, 95% CI: 804.72 to 3,278.38; p=0.001) more expensive than early-week surgeries.

Conclusions

At our institution, patients undergoing one- to three-level lumbar fusion surgery on Thursday or Friday had a higher risk of non-home discharge, 30-day readmission, and incurred higher cost than those having early-week surgery. Further research is needed to elucidate the reasons for these findings and to evaluate interventions aimed at improving outcomes for patients undergoing surgery later in the week.

## Introduction

Lumbar fusion surgery for degenerative spinal conditions is responsible for significant healthcare expenditures in the United States, and the volume of surgeries is increasing over time [1-3]. Traditionally, the predominant form of healthcare reimbursement in the United States has been fee-for-service, though this is becoming increasingly unsustainable as healthcare costs rise [4,5]. With the advent of initiatives such as the Affordable Care Act and bundled payment models, there is an increased emphasis on value and quality-based reimbursements, which place more financial responsibility on individual providers and healthcare systems [5-7]. This in turn has caused increased focus on factors that improve outcomes and reduce overall costs [8,9].

One factor that has not been well studied is the effect of surgical day of the week on patient outcomes. A number of studies have identified an association between weekend surgeries and adverse patient outcomes and increased cost, possibly due to the reduced availability of ancillary staff on the weekend [7,8,10,11]. Fewer studies have looked at surgeries performed Monday through Friday and the effect this has on length of stay (LOS), discharge disposition, patient outcomes, and cost. A number of studies in the total joint arthroplasty literature have identified an association between surgical day of the week and length of stay, with later-week surgeries experiencing longer LOS [9,12,13]. In the spine surgery literature, the association has been more mixed, with some studies identifying longer LOS with later-week surgeries and some studies not showing this same association [5,8,14,15].

The purpose of this study is to compare LOS, patient outcomes, and hospital costs between patients having surgery early in the week (Monday through Wednesday) and later in the week (Thursday and Friday). We hypothesize that patients undergoing surgery later in the week will experience worse outcomes when compared to patients undergoing surgery early in the week.

## Materials and methods

This study was deemed exempt as a retrospective review of existing medical records by the institutional review board.

Study population

A retrospective review of 771 patients undergoing one- to three-level lumbar fusion from December 2020 to December 2023 was performed. All patients included had surgery on Monday, Tuesday, Wednesday, Thursday or Friday; 56 patients having surgery on Saturday or Sunday were excluded. All surgeries were performed by one of six board-certified orthopedic spine or neurosurgeons at a single institution.

Study outcomes

The outcomes of interest included minutes in the operating room (OR), LOS in hours and days, total cost, non-home discharge, 30-day emergency department (ED) return, days to ED return, reason for ED return (medical or surgical), 30-day readmission, days to readmission, reason for readmission (medical or surgical), 30-day return to the OR, days to return to OR, and one-year revision fusion. Direct cost of care for each initial hospitalization was assessed using activity-based costing (Costflex Inc., Mobile, AL, USA). Total direct variable cost (i.e. the total specific cost of caring for the patient), labor cost, supply cost, and drug cost were each evaluated.

Independent variables

The independent variables of interest included age, sex, body mass index (BMI), race, American Society of Anesthesiologist (ASA) score, Charlson Comorbidity Index (CCI), number of operative levels, procedure performed (posterolateral fusion [PLF], posterior or transforaminal interbody fusion [PLIF/TLIF], lateral or extreme lateral interbody fusion [LLIF/XLIF]) and inpatient/outpatient procedure status.

Statistical analysis

Patients were grouped by their surgical day of week; those with surgery Monday, Tuesday or Wednesday were early week and those with surgery on Thursday or Friday were late week. Univariate analyses including chi-square tests and independent sample t-tests were performed to determine differences in patient and procedure details, and postoperative outcomes between those who had surgery early in the week and those who had surgery late in the week. Multivariate linear and logistic regression were used to assess the effect of late-week surgery on outcomes controlling for sex, BMI, CCI, number of operative levels and procedure performed. Bar graphs were used to display average LOS and rates of non-home discharge by surgical day of the week. All statistical analyses were performed using R Studio (Version 4.2.2; Posit PBC, Boston, MA, USA). Statistical significance was assessed at p<0.05.

Source of funding

This study did not receive any funding.

## Results

Of the 715 patients included, 158 (22.1%) had surgery on a Monday, 159 (22.2%) had surgery on a Tuesday, 169 (23.6%) had surgery on a Wednesday, 124 (17.3%) had surgery on a Thursday, and 105 (14.7%) had surgery on a Friday. There were 486 (68.0%) early-week surgeries and 229 (32.0%) late-week surgeries. Overall, there were no differences in age, sex, BMI, race, ASA scores, CCI scores, number of operative levels or inpatient/outpatient status between early- and late-week surgeries. Interbody fusion procedures (PLIF/TLIF) accounted for a greater portion of late-week surgeries (65.9 vs. 44.4%, p<0.001) (Table 1).

**Table 1:** Patient and Procedure Details by Surgical Time of the Week P-Value < 0.05 in bold; all data presented as mean ± SD or n (%); BMI, body mass index; ASA, American Society of Anesthesiologists; CCI, Charlson Comorbidity Index; PLF, posterior lumbar fusion; PLIF, posterior lumbar interbody fusion; TLIF, transforaminal lumbar interbody fusion; XLIF, extreme lateral interbody fusion; LLIF, lateral lumbar interbody fusion

Patient and Procedure Details	Early Week (n=486)	Late Week (n=229)	P-Value
Age, years (mean ± SD)	62.8 ± 12.5	62.0 ± 12.1	0.444
Sex (n (%))			0.176
Female	274 (56.4)	116 (50.7)	
Male	212 (436.)	113 (49.3)	
BMI, kg/m^2 ^(mean ± SD)	30.8 ± 5.6	31.5 ± 5.9	0.112
Non-White Race (n (%))	98 (20.2)	38 (16.6)	0.302
ASA Score 3+ (n (%))	257 (52.9)	128 (55.9)	0.500
CCI Score (mean ± SD)	2.4 ± 1.6	2.3 ± 1.6	0.272
# Levels (mean ± SD)	1.9 ± 0.7	1.8 ± 0.8	0.379
Procedure (n (%))			
PLF	261 (53.7)	77 (33.6)	<0.001
PLIF/TLIF	216 (44.4)	151 (65.9)	<0.001
XLIF/LLIF	9 (1.9)	1 (0.4)	0.245
Inpatient/Outpatient Procedure (n (%))			0.935
Inpatient	294 (60.5)	140 (61.1)	
Outpatient	192 (39.5)	89 (38.9)	

Postoperatively, there was no difference in operative time, length of stay, discharge destination, 30-day ED return, 30-day readmission, 30-day return to the OR, or one-year revision fusion between groups. However, late-week surgeries were, on average, $3,697 USD more expensive than early-week surgeries ($26,506 ± 8,913 vs. $22,809 ± 9,598 USD; p<0.001) (Table 2).

**Table 2:** Outcomes by Surgical Time of the Week P-Value < 0.05 in bold; all data presented as mean ± SD or n (%); OR, operating room, USD, United States dollars; LOS, length of stay; ED, emergency department

Outcome	Early Week (n=486)	Late Week (n=229)	P-Value
OR Time, minutes (mean ± SD)	224.2 ± 66.6	225.3 ± 51.6	0.812
Total Cost, $ USD (mean ± SD)	22,809 ± 9,598	26,506 ± 8,913	<0.001
LOS, Hours (mean ± SD)	60.8 ± 61.1	63.8 ± 51.3	0.487
LOS, Days (mean ± SD)	2.2 ± 2.5	2.3 ± 2.1	0.513
Non-Home Discharge (n (%))	23 (4.7)	20 (8.7)	0.053
30 Day ED Return (n (%))	27 (5.6)	8 (3.5)	0.314
Days to ED Return (mean ± SD)	14.5 ± 9.3	13.4 ±7.0	0.703
Reason for ED Return (n (%))			1
Medical	14 (51.9)	4 (50.0)	
Surgical	13 (48.1)	4 (50.0)	
30 Day Readmission (n (%))	15 (3.1)	14 (6.1)	0.087
Days to Readmission (mean ± SD)	16.0 ± 8.4	14.2 ± 8.1	0.519
Reason for Readmission (n (%))			0.798
Medical	6 (40.0)	4 (28.6)	
Surgical	9 (60.0)	10 (71.4)	
30 Day Return to OR (n (%))	9 (1.5)	10 (3.7)	0.062
Days to Return to OR (mean ± SD)	14.1 ± 10.2	14.3 ± 7.7	0.965
1 Year Revision Fusion (n (%))	13 (2.7)	6 (2.6)	1

After controlling for sex, BMI, CCI score, number of operative levels and procedure performed, late-week surgeries were 2.47 times more likely to have a non-home discharge (OR: 2.47, 95% CI: 1.24 to 4.95; p=0.010) and 2.19 times more likely to have a 30-day readmission (OR: 2.19, 95% CI:1.01 to 4.74; p=0.044) (Table 3).

**Table 3:** Multivariate Linear and Logistic Regression: Risk Adjusted Outcomes by Time of Week Controlling for sex, BMI, CCI, # of levels, and procedure; P-Value < 0.05 in bold; OR, operating room; LOS, length of stay; ED, emergency department; BMI, body mass index; CCI, Charlson Comorbidity Index

Outcome	Late Week β/OR	95% OR CI	P-Value
OR Time, minutes	-6.21	-15.17 to 2.74	0.173
LOS, hours	3.25	-5.57 to 12.08	0.470
LOS, days	0.13	-0.24 to 0.50	0.489
Non-Home Discharge	2.47	1.24 to 4.95	0.010
30 Day ED Return	0.67	0.28 to 1.46	0.341
30 Day Readmission	2.19	1.01 to 4.74	0.044
30 Day Return to OR	2.39	0.93 to 6.24	0.069
Reoperation	0.66	0.27 to 1.49	0.342
1 Year Revision Fusion	0.79	027 to 2.09	0.647

Additionally, after risk adjustment, late-week surgeries were $2,041.55 USD (β: 2,041.55, 95% CI: 804.72 to 3,278.38; p=0.001) more expensive than early-week surgeries overall. Examination of specific cost components revealed that supply costs were approximately $1,895 USD higher in late-week surgeries (β: 1894.62, 95% CI: 883.78 to 2905.46, p<0.001); no significant differences in labor or drug costs were observed between groups (Table 4).

**Table 4:** Multivariate Linear Regression: Risk Adjusted Cost Components by Time of Week Controlling for sex, BMI, Charlson Comorbidity Index (CCI), # of levels, and procedure; P-Value < 0.05 in bold; all costs in USD ($)

Cost Component	Late Week β	95% OR CI	P-Value
Total Cost	2,041.55	804.72 to 3,278.38	0.001
Labor Cost	87.29	-358.68 to 533.26	0.701
Supply Cost	1894.62	883.78 to 2905.46	<0.001
Drug Cost	-4.53	-53.47 to 44.41	0.856

**Figure 1:**
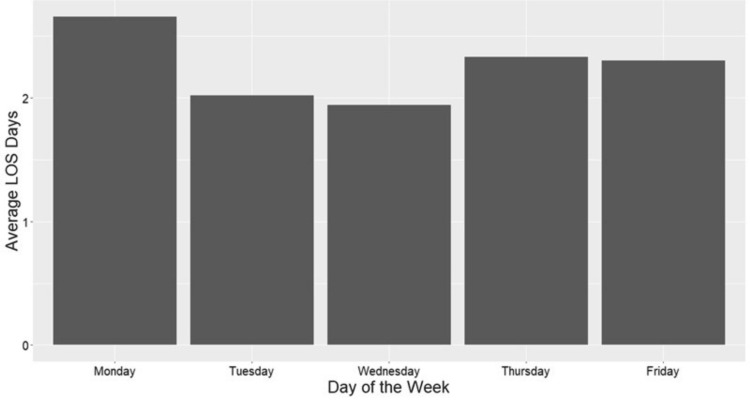
Average LOS by Surgical Day of the Week LOS, length of stay

Overall, Monday surgeries had the longest average LOS (3.3 ± 3.6 days) followed by Thursday (2.6 ± 2.4 days) and Friday (2.5± 2.2 days), while Wednesday (2.3 ± 2.0 days) and Tuesday (2.3 ± 1.7 days) had the shortest average LOS (Figure 1).

**Figure 2:**
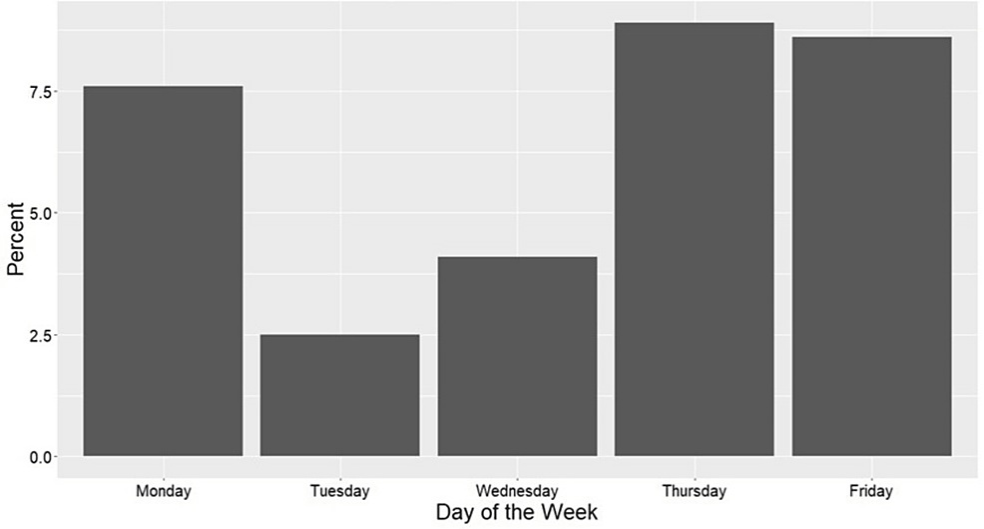
Percent Non-Home Discharge by Surgical Day of the Week

Similarly, Thursday surgeries had the highest rate of non-home discharge at 8.9% (11 patients) followed by Friday at 8.6% (9 patients) and Monday at 7.6% (12 patients), while Wednesday at 4.1% (7 patients) and Tuesday at 2.5% (4 patients) had the lowest rate of non-home discharges (Figure 2).

## Discussion

With the current emphasis in healthcare of cost containment and quality improvement, the effect of surgical day of week on postoperative outcomes is unclear. This study examined the effect of surgical day of week in patients undergoing one- to three-level lumbar fusions on cost and postoperative outcomes. Patients who underwent surgery in the latter half of the week had increased hospital costs in both univariate and multivariate analysis. These patients were also nearly 2.5 times more likely to experience non-home discharge and more than two times more likely to experience readmission in the first 30 days after surgery.

A number of studies in the orthopedic and spine surgery literature have found that patients undergoing surgery later in the week were more likely to experience longer lengths of stay when compared with patients undergoing surgery earlier in the week [7-9,15-17]. In a recent study by Heard et al., patients undergoing lumbar fusion later in the week (Thursday or Friday) had an increased length of stay that was nearly half a day longer than patients undergoing surgery earlier in the week [8]. These patients also experienced a longer wait for inpatient physical therapy and an increased number of neurologic complications, both of which may have influenced the length of stay [8]. A study by Salas-Vega et al. had a similar finding in patients undergoing lumbar laminectomy; patients who had surgery on a Thursday or Friday had a length of stay that was almost half a day longer than patients having surgery earlier in the week, and the LOS discrepancy was increased when considering patients discharging to a skilled nursing facility (SNF) or patients who experienced postoperative complications [17]. A study by Sivaganesan et al. examined LOS in patients undergoing lumbar surgery and found that patients having lumbar fusions or discharging to a SNF had a longer length of stay when surgery was performed later in the week [18]. A number of studies surmised that the increased length of stay in patients having surgery later in the week was due in part to the reduced ancillary staff availability on the weekends [15,18,19]. Contrary to these studies, our study found no significant differences in length of stay between early-week and late-week surgeries, in either univariate or multivariate analysis. While the scope of this study did not allow us to elucidate the cause, our institution does have a coordinated spine surgical program with highly protocolized discharge processes, which may mitigate the reduced access to ancillary staff over the weekends.

Hospital readmission following spinal surgery is an unfavorable outcome and leads to increased costs for the patient and hospital; readmission is also used as a quality metric by the Centers for Medicare and Medicaid Services (CMS) and may affect hospital reimbursement [8,11,20]. A study by Rosenberg et al. found that patients admitted for thoracolumbar fusion on the weekend were more than twice as likely to be readmitted to the hospital within 30 days [11]. Graham et al. found a similar risk of 30-day readmission in patients undergoing adult spinal deformity surgery when comparing early-week surgeries to late-week surgeries, while also noting no difference in LOS or other complications [14]. Similar to the study by Graham et al., our study found patients undergoing late-week surgery were more than twice as likely to be readmitted within 30 days, but there was no difference in LOS or in other complications. We initially hypothesized that late-week surgeries discharging over the weekend may experience higher ED return and readmission rates immediately following discharge given the lack of clinic access during this time. However, our finding that ED returns and readmissions occurred at approximately two weeks postoperatively, on average, in both early- and late-week surgeries contradicts this assertion. In light of this finding, and the similar mix of medical and surgical reasons for hospital returns between groups, further research is needed to evaluate potential reasons for the increased risk of readmission observed in late-week surgeries.

Cost of care for surgical admissions is a widely scrutinized topic and a variety of methods are being discussed to reduce cost and improve efficiency and value in healthcare [5,8]. Length of stay for orthopedic surgical admissions has been shown in a number of studies to increase the overall cost associated with that admission [7,9,17]. Boylan et al. found that patients undergoing total knee arthroplasty later in the week had both an increased LOS and increased cost when compared to patients undergoing surgery earlier in the week [7]. Salas-Vega et al. found that patients undergoing lumbar laminectomy later in the week also experienced a longer LOS and greater hospital costs, although the patients did not have increased underlying health risks [17]. In contrast, Khechen et al. found no difference between early- and late-week surgeries for either LOS or cost in patients undergoing anterior cervical fusion surgery [5]. While patients in this study had no increased LOS between early-week and late-week surgeries, we did note an increased cost of approximately $2000 USD for patients undergoing surgery on Thursday or Friday. However, this cost difference was driven primarily by increased supply costs, even after adjusting for procedure type and number of levels treated. It is therefore likely that the implant preferences of surgeons routinely operating later in the week, rather than differences in care delivery, resulted in the increased cost observed.

The results of this study do need to be considered in light of the potential limitations. It is a retrospective study from a single institution and the results may not be generalizable to a wider patient population. Further, the observational nature of the study precludes our ability to assess the causes of the trends observed. The retrospective nature of the data collection also inherently introduces the potential for selection bias. The day of week data may also be influenced by the individual surgeon’s prearranged block time, and this was not included in our analysis. We did attempt to control for some of this variability in the multivariate analysis by controlling for number of levels and procedure type. Finally, the cost analysis performed in this study evaluated only variable costs during hospitalization for the initial surgery. Given the higher rates of SNF utilization and readmissions (both of which are known high-cost resources) among late-week surgeries in this study, further evaluation of episode of care costs by day of week is needed [21-23].

## Conclusions

At our institution, patients undergoing one- to three-level lumbar fusion surgery on Thursday or Friday had a higher risk of non-home discharge and 30-day readmission when compared to patients having surgery on Monday, Tuesday, or Wednesday. In addition, these patients had increased hospital costs, primarily due to increased supply costs. Further research is needed to elucidate the reasons for these findings and to evaluate interventions aimed at improving outcomes for patients undergoing surgery later in the week.
